# Diversity of Ginsenoside Profiles Produced by Various Processing Technologies

**DOI:** 10.3390/molecules25194390

**Published:** 2020-09-24

**Authors:** Xiang Min Piao, Yue Huo, Jong Pyo Kang, Ramya Mathiyalagan, Hao Zhang, Dong Uk Yang, Mia Kim, Deok Chun Yang, Se Chan Kang, Ying Ping Wang

**Affiliations:** 1 State Local Joint Engineering Research Center of Ginseng Breeding and Application, Jilin Agriculture University, Changchun 130118, China; pxm52_@163.com (X.M.P.); zhanghaoscience@163.com (H.Z.); dcyang@khu.ac.kr (D.C.Y.); 2 Graduate School of Biotechnology, College of Life Sciences, Kyung Hee University, Yongin si, Gyeonggi do 17104, Korea; huoyue0214@gmail.com (Y.H.); kangjongpyo@naver.com (J.P.K.); ramyabinfo@gmail.com (R.M.); rudckfeo23@naver.com (D.U.Y.); 3 Institute of Special Wild Economic Animals and Plants, Chinese Academy of Agricultural Sciences, Changchun 130112, China; 4 Department of Cardiovascular and Neurologic Diseases, College of Korea Medicine, Kyung Hee University, Seoul 100011, Korea; hyuntemia@hanmail.net

**Keywords:** ginsenoside, processed ginseng, physical, chemical, dehydration, biotransformation, glycosyltransferases

## Abstract

Ginseng is a traditional medicinal herb commonly consumed world-wide owing to its unique family of saponins called ginsenosides. The absorption and bioavailability of ginsenosides mainly depend on an individual’s gastrointestinal bioconversion abilities. There is a need to improve ginseng processing to predictably increase the pharmacologically active of ginsenosides. Various types of ginseng, such as fresh, white, steamed, acid-processed, and fermented ginsengs, are available. The various ginseng processing methods produce a range ginsenoside compositions with diverse pharmacological properties. This review is intended to summarize the properties of the ginsenosides found in different *Panax* species as well as the different processing methods. The sugar moiety attached to the C–3, C–6, or C–20 deglycosylated to produce minor ginsenosides, such as Rb1, Rb2, Rc, Rd→Rg3, F2, Rh2; Re, Rf→Rg1, Rg2, F1, Rh1. The malonyl-Rb1, Rb2, Rc, and Rd were demalonylated into ginsenoside Rb1, Rb2, Rc, and Rd by dehydration. Dehydration also produces minor ginsenosides such as Rg3→Rk1, Rg5, Rz1; Rh2→Rk2, Rh3; Rh1→Rh4, Rk3; Rg2→Rg6, F4; Rs3→Rs4, Rs5; Rf→Rg9, Rg10. Acetylation of several ginsenosides may generate acetylated ginsenosides Rg5, Rk1, Rh4, Rk3, Rs4, Rs5, Rs6, and Rs7. Acid processing methods produces Rh1→Rk3, Rh4; Rh2→Rk1, Rg5; Rg3→Rk2, Rh3; Re, Rf, Rg2→F1, Rh1, Rf2, Rf3, Rg6, F4, Rg9. Alkaline produces Rh16, Rh3, Rh1, F4, Rk1, ginsenoslaloside-I, 20(S)-ginsenoside-Rh1-60-acetate, 20(R)-ginsenoside Rh19, zingibroside-R1 through hydrolysis, hydration addition reactions, and dehydration. Moreover, biological processing of ginseng generates the minor ginsenosides of Rg3, F2, Rh2, CK, Rh1, Mc, compound O, compound Y through hydrolysis reactions, and synthetic ginsenosides Rd12 and Ia are produced through glycosylation. This review with respect to the properties of particular ginsenosides could serve to increase the utilization of ginseng in agricultural products, food, dietary supplements, health supplements, and medicines, and may also spur future development of novel highly functional ginseng products through a combination of various processing methods.

## 1. Introduction

**Figure 1:**
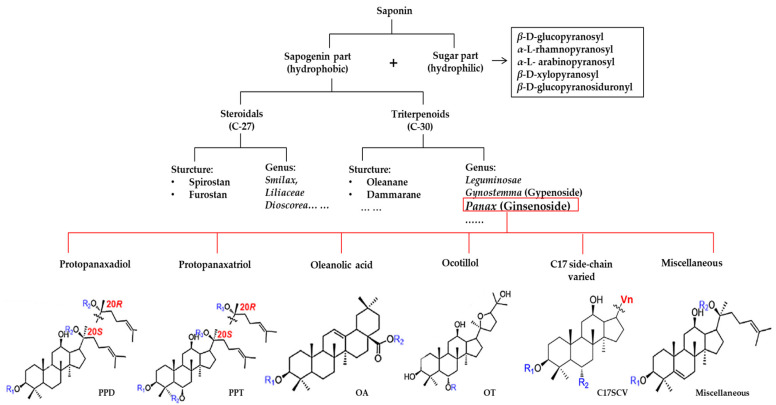
Various structure types of *Panax* ginsenosides.

Herbal products are widely used in medicine, food production, and other fields due to their various active compounds and beneficial effects. In addition to the well-known active proteins, polysaccharides, polyphenols, and flavonoids, saponins are also very important active substances in herbs. Saponins belong to the group of plant glycosides widely distributed in more than 100 families of both wild and cultivated plants and in some marine organisms [1]. Saponins consist of a polycyclic aglycones attached to one or more hydrophilic (water-soluble) sugar side chains. The hydrophobic (fat-soluble) aglycone moiety, which is also called sapogenin, is either a steroid (C–27) or a triterpene (C–30) [2]. Steroidal saponin are typically contain 27 carbon atoms forming the core: Spirostan and furostan structures, which are predominantly found in monocotyledons (such as *Liliaceae, Smilax,* or *Dioscorea*) [1,3,4]. In contrast, triterpenoid saponins are common metabolites in secondary dicotyledons (such as *Leguminosae,* or *Panax*). Triterpenes belong to a large group of compounds arranged in four-(Dammarane) or five-ring (Oleanane) configurations of 30 carbons [5]. Ginsenosides are triterpenoid saponins with a four-ring skeleton structure and are unique to ginseng species belonging to the genus *Panax*. The ginsenosides identified to date are classified as the protopanaxatriol type (PPT), protopanaxadiol type (PPD), oleanolic acid type (OA), ocotillol type (OT), C17 side-chain varied (C17SCV), and miscellaneous subtypes according to their known structures (Figure 1) [6]. The PPD type consists of an aglycone with a dammarane skeleton and sugar moieties attached to the β-OH at C–3 and/or C–20. The PPT type consists of an aglycone with a dammarane skeleton and sugar moieties attached to the α-OH at C–6 and/or β-OH at C–20. The oleanane group consists of OA and OT types: The OA type consists of a pentacyclic structure with an aglycone oleanolic acid such as ginsenoside Ro; the OT has an epoxy ring at C–20, and includes majonoside R2 and the pseudoginsenoside F11 (p-F11) [7]. Moreover, the substituents within the C17 side chains often undergo oxidation, reduction, cyclization, and epimerization, contributing additional diversity in ginsenoside chemical structure [8,9].

*Panax* species have long been important sources of natural medicines due to their abundant ginsenosides [9]. Ginseng belongs to the genus *Panax* in the family Araliaceae. Of the 17 different species assigned to this genus, the most widely used *Panax* species is *P. ginseng* (Korean ginseng), which is considered one of the most valuable medicinal herbs in traditional Asian medicine. Another member of the genus is *P. quinquefolius* (American ginseng), which was valued by the American Indians long before the arrival of Europeans in the New World; American ginseng has been cultivated in North America for medicinal purposes since the eighteenth century. Other *Panax* species commonly used in herbal medicine are *P. japonicus* (Japanese ginseng), *P. notoginseng* (Chinese ginseng), *P. vietnamensis* (Vietnamese ginseng), *P. omeiensis* (Omei ginseng), *P. pseudoginseng* (Himalayan ginseng), *P. zingiberensis* (ginger ginseng), *P. stipuleanatus* (Pingpien ginseng), *P. trifolius* (dwarf ginseng), *P. wangianus* (narrow-leaved pseudoginseng), *P. bipinnatifidus* (feather-leaf bamboo ginseng), *P. variabilis*, *P. sokpayensis*, *P. assamicus*, *P. shangianus*, and *P. sinensis* [10,11].

Many different processing techniques are employed, ranging from fresh ginseng (right out of the field with simple washing) to various physical (drying, boiling, steaming, heating, sulfur-fumigation, microwaving, and puffing), chemical (acid and alkaline treatments), and biological (fermentation using microbial or enzymes) processes [12,13,14]. There are a large number of reports describing the processing conditions and main methods for ginsenoside analysis [15,16]. However, there have been few reviews addressing the diversity of ginsenosides found in various ginsengs subjected to different processing methods. Such information may allow better use of the various ginsengs and provide clues to direct future research into the biological functions of ginsenosides. Therefore, this review summarizes the distribution of ginsenosides in different ginseng species and processed products, and aims to improve the utilization of ginseng and particular ginsenosides in agricultural products, food, dietary supplements, health supplements, and medicines.

## 2. Comparison of the Major Ginsenosides in Various *Panax* Species

Most of the pharmacological and bioactive effects of ginseng are produced by various ginsenosides. Ginsenosides exhibit diverse biological activities, including anti-diabetic, anti-aging, anti-carcinogenic, anti-fatigue, anti-pyretic, and anti-stress activities, and are also known to promote DNA, RNA, and protein synthesis [17,18,19,20,21,22]. Therefore, ginsenosides are recognized as a key index for quality evaluation of ginseng. The distribution of ginsenosides varies among different *Panax* species. Among ginsenosides isolated from ginseng plants to date, Rb1, Rb2, Rc, Rd, Re, Rf, and Rg1 typically constitute more than 90% of the total ginsenosides and are usually regarded as the major ginsenosides [23]. The distribution of these major ginsenosides varies significantly due to environmental effects, including soil fertility, temperature, light, and humidity [24]. Therefore, accurate determinations of the type and amount of ginsenosides in different *Panax* species are not only important for the pharmacological evaluation of various ginsengs, but also when assessing the quality of ginseng cultivated in different countries. 

Recently, 17 ginseng species in the genus *Panax* were described by Zhang et al. (2020) [11], and the results are summarized in Table 1. Major ginsenosides (including Rb1, Rb2, Rc, Rd, Re, Rf, and Rg1) are naturally present in most ginseng plants, however the distribution and concentrations of the major ginsenosides differ in each *Panax* species. Park et al. (2017) [25] reported that six major ginsenosides (Rb1, Rb2, Rc, Rd, Re, and Rg1) were found to comprise 90% of the total ginsenoside content of *P. ginseng*, and Chen et al. (2019) [24] used the six ginsenosides to evaluate ginsenoside abundance in *P. ginseng* from different regions. The major ginsenosides are also present in other ginseng species at varying concentrations. The Rb1:Rg1 ratio is commonly used to assess the differences between ginsengs: Rb1:Rg1 ratios between 1 and 3 are typical for *P. ginseng* and *P. notoginseng,* while ratios around 10 or greater are characteristic of *P. quinquefolius* [13]. In addition, several researchers have identified potential marker ginsenosides that could discriminate among ginseng species: For example, Yang et al. (2016) [26] found that ginsenoside Rf and Rs_1_ were unique to *P. ginseng,* whereas pseudoginsenoside-F11 (p-F11) was only present in *P. quinquefolius*; four characteristic markers for *P. notoginseng* (Ra3) and *P. quinquefolius* (Ro and p-F11) can be used to differentiate American ginseng and Notoginseng; Rf, Re, Rg1, Rc, Rb2, and Rd are abundant in *P. ginseng*; Re, Rb1, and Rd are rich in *P. quinquefolius*; and noto-R1, Rg1, and Rd are plentiful in *P. notoginseng* [27].

In addition to *P. ginseng, P. notoginseng,* and *P. quinquefolius*, the activity and ginsenoside composition of several other species available worldwide have also been investigated. *P. japonicas* Meyer (Japanese ginseng or Ye-sanchi, wild grown throughout Japan and the south of Yunnan province, China) has been used to promote the functional activity of the stomach and as a tonic, antitussive, anti-inflammatory, and hemostatic agent. Several reports have indicated that ocotillol type saponins (Yesanchinosides) and majonoside R2 were the major compounds in *P. japonicas,* and major ginsenosides (Rc, Rg1, Rd, and Rb3) and notoginsenosides (R1, R4) were also present [32,33]. *P. vietnamensis* (Vietnam ginseng, a wild ginseng species found at Mount Ngoc Linh in Central Vietnam) with similar activity to *P. ginseng*, is used for enhancement of physical strength and treatment of many diseases. The main saponins of *P. vietnamensis* were not only ginsenoside Rb1, Rd, Re, and Rg1, but also OT saponins (majonoside R1, R2, and vinaginsenoside R1 and R2). In addition, majonoside R2 is present at high concentrations in *P. vietnamensis* and appears to be responsible for its remarkable pharmacological effects, and is used to distinguish *P. vietnamensis* from other species [36,37]. *P. zingiberensis* is widely used in folk medicines in China and Myanmar to strengthen the immune response and provide cardiovascular protection [41]. It exhibits anticancer activity and prevents platelet aggregation because of the presence of a large number of OA-type ginsenosides, such as ginsenoside Ro, chikusetsu saponin-IV (CS-IV) and CS-Iva; PPT-type ginsenosides (Rg1, Re, and F3) are also present in *P. zingiberensis* at higher concentrations than the PPD-type ginsenosides [39,42]. Zingibroside R1 has also been detected in *P. zingiberensis* and has a structure similar to the OA-type ginsenoside Ro, although it lacks glucose at the C28 position [39]. *P. stipuleanatus* Tsai et Feng is an herb grown in Southeast Yunnan, China, and North Vietnam, which contains OA-type triterpenoids (spinasaponin A methyl ester, stipuleanoside R1 and R2, pesudoginsenoside RP_1_ and RT_1_, CS-IVa) as the major components [44,45].

In addition to the above well-recognized *Panax* species, several species of the genus *Panax,* such as *P. bipinnatifidus* and *P. sokpayensis*, flourish in the Himalayan region. Tung et al. (2011) reported that ten OA-type saponins were isolated from *P. bipinnatifidus,* including bifinosides A–C, CS–IVa, pseudoginsenoside RP_1_ and RT_1_, stipuleanoside R1 and R2 [46]. Dammarane-type major ginsenosides (Rg1, Rg2, Re, Rd, and Rb1) are also present at higher concentrations in *P. bipinnatifidus* compared with *P. sokpayensis* (containing Rg1, Rg2, Re, Rd, Rb1, and Rb2), although Rc has not been detected in either *Panax* species [47].

## 3. Variations in Ginsenoside Compositions due to Different Processing Technologies

**Figure 2:**
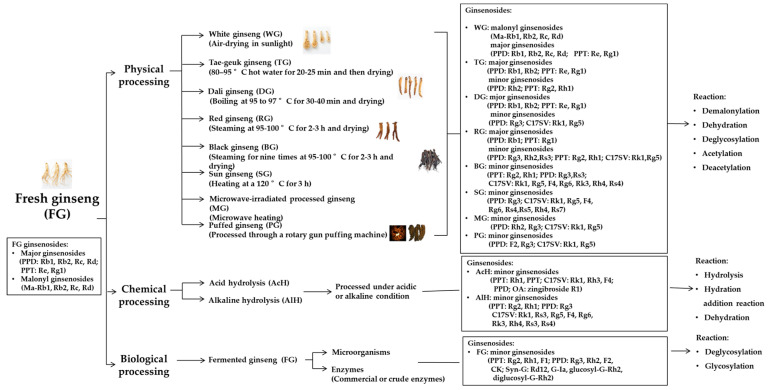
Various types of ginseng processed through different methods. PPT, protopanaxatriol; PPD, protopanaxadiol; C17SCV, C17 side-chain varied; OA, oleanolic acid; Syn-G, synthetic ginsenoside; G, ginsenoside; Ma, malonyl.

In order to increase the pharmacological effects of ginseng and related products and reduce their side-effects or toxicity, many processing approaches have been developed. The particular processing technique selected may play an important role in the application and utilization of ginseng, as the processing conditions produce variations in the ginsenoside constituents [62]. The various ginseng processing technologies, such as physical, chemical, biological treatments, are outlined in Figure 2.

### 3.1. Ginsenoside Variations in Physically Processed Ginseng 

Physical methods are widely used to process fresh ginseng into consumable products, because fresh ginseng (non-processed ginseng after harvest) may easily deteriorate due to a high water content (70–80%) and the presence of soil microorganisms. In addition to commonly employed dehydration and steaming methods, other physical processing methods include sulfur fumigation and microwave processing. The following sections address the variations in the ginsenoside profiles of various products obtained using physical processing methods, as described in recent reports.

#### 3.1.1. Fresh Ginseng (FG) and White Ginseng (WG)

White ginseng (WG) is produced by air-drying fresh ginseng (FG) in sunlight until it reaches low water concentrations (≤12%), and is a commonly consumed product available in herbal markets. Major ginsenosides are abundant in both ginsengs with a slightly difference in content of each ginsenoside. In addition to the major ginsenosides (Rb1, Rb2, Rc, Rd, Re, Rf, and Rg1), another important group of natural ginsenosides, the malonyl ginsenosides, are also present in both FG and WG. Malonyl ginsenosides contain a malonyl residue attached to the glucose unit of the corresponding major ginsenoside, such as Rb1, Rb2, Rc, or Rd, to form malonyl-Rb1, malonyl-Rb2, malonyl-Rc, or malonyl-Rd; the modified ginsenosides are unstable and can demalonylate to form the corresponding ginsenosides upon processing [63,64]. Compared with FG, the concentrations of total and malonyl ginsenosides in WG are reduced after processing by air-drying. The ginsenosides Rg1, Re, Rb1, Rc, and Rb2 are abundant in fresh ginseng; however, the concentrations of Rb1 and Rg1 are increased in WG [14,65]. Although air-drying FG alters ginsenoside composition in the resulting WG, the rare ginsenosides are barely detectable in both FW and WG.

#### 3.1.2. Tae-Geuk Ginseng (TG) and Dali Ginseng (DG)

Tae-geuk ginseng (TG) could be described as an intermediate product between red ginseng and white ginseng, as it is produced by heating fresh ginseng in 80–95 °C water for 20–25 min and then drying it. The color of the resulting product is different from that of WG. Several studies [66,67] have indicated that the key saponin compounds of tae-geuk ginseng are PPT (Rh2, Re, Rf, Rh1, and Rg1) and PPD (Rc, Rb2, Rg3, and Rb1). Another study found Re, Rb1, Rb2, Rg2, and Rh1 were the main ginsenosides in TG [68]. Compared with FW and WG, several rare ginsenosides (such as Rh1, Rh2, Rg2, and Rg3) are present at greater concentrations in TG following processing.

There is a ginseng processed using a method similar to that of TG processing, called Dali ginseng (DG). Dali ginseng, also called boiled ginseng in China, is processed by boiling fresh ginseng at 95–97 °C for 30–40 min and then drying the product [69]. The medicinal value of DG is thought to be similar to WG, but DG also has a mild “warming effect”. Several studies have found that the malonyl ginsenosides abundant in WG are not present in DG after high-temperature processing. The Rk1 and Rg5 dehydration products of 20(S)—Rg3 are found in DG, but not in WG, while noto—R1, Rs5, and Rs4 are detected only in DG and not in other ginsengs [70].

#### 3.1.3. Red Ginseng (RG) and Black Ginseng (BG) 

Instead of processing by air-drying and heating for short periods, red ginseng is prepared by steaming fresh ginseng at 95–100 °C for 2–3 h before drying. The color of the ginseng changes from white/yellow to red after steaming [71,72]. Red ginseng is reportedly more pharmacologically active than WG and is commonly consumed around the world. The improved biological activity of red ginseng is due to changes in the ginsenoside composition following steaming [73]. Malonyl and major ginsenosides that are present at high concentrations in FW and WG are absent or reduced in RG, but Rb1 is present at high concentrations, possibly due to the dramatic loss of malonyl-Rb1 after steaming [72]. After steaming, the concentrations of ginsenosides Rg2, Rg3, Rh2, and Rh1 in RG are greater than in TG or DG, and the rare ginsenosides Rk1, Rs3, and Rg5 are also present in RG. The present of rare ginsenosides promotes pharmacologically activities more than the above ginsengs [67]. 

Black ginseng, as a promoted product of RG, is produced by steaming raw ginseng nine times at 95–100 °C for 2–3 h and then dehydrating the resulting product. Because of the different processing method, the color of ginseng changes to black and special ginsenosides are produced in addition to the main ginsenosides found in FG and WG. The major ginsenosides Rg1, Re, Rb1, Rc, Rb2, and Rd are considerably reduced, while the concentrations of the minor ginsenosides Rg2, Rg3, Rh1, Rk1, Rs3, and Rg5 in BG are greater than in RG. The notable structural changes produced during the steam cycles are hydrolysis of the sugar moieties at C–3, C–6 or C–20 and subsequent dehydration at C–20 [74,75]. In addition, BG contains some ginsenosides (F4, Rg6, Rk3, Rh4, Rs3, and Rs4) that are absent or present only in trace amounts in RG and exhibits more potent biological activity than WG or RG [76,77]. 

#### 3.1.4. Sun Ginseng (SG)

To enhance the yield of the specific ginsenosides in RG and BG, a method of steaming raw ginseng at a higher temperature (120 °C) for 3 h has been developed. Heat-processed ginseng, designated as sun ginseng (SG), has been found to possess multiple biological activities, such as radical scavenging, anti-tumor, vasorelaxation, and anti-diabetes activities [78,79,80]. Sun ginseng also possesses greater NO-scavenging activity than other steamed ginsengs [73]. After heating at high temperature, the major ginsenosides (such as Rb1, Re, Rb2, Rc, Rd, Rg1, and malonyl-ginsenosides) are not detectable in SG or are present only at very low concentrations; however, the concentrations of the rare ginsenosides Rg3, Rg5, Rh4, and Rk1 are greatly increased, and several new acetylated ginsenosides (Rs4, Rs5, Rs6 and Rs7) have also been isolated from SG [28,81]. Moreover, unlike the conversion of PPT- and PPD-type ginsenosides during the heating process, the ocotillol-type saponins, which have no glycosyl moiety at the C-20 position, are relatively stable upon heating [37].

#### 3.1.5. Sulfur-Fumigated Ginseng

In addition to air-drying or heating to process ginseng post-harvest, sulfur fumigation is also used. In this method, slices of ginseng and sulfur powder are placed in a desiccator in the lower and upper layers at 25 °C for 12 h. Sulfur-fumigation processing may reduce the concentrations of major ginsenosides (Rg1, Re, Rc, Rb2, and Rd) and produce some minor ginsenosides, such as Rh2 and Rg5, through hydrolysis, dehydration, and decarboxylation [82]. However, sulfur fumigation may also produce some hazardous substances, and several reports have recommended that sulfur fumigation not be used for post-harvest processing of ginseng [83,84].

#### 3.1.6. Microwave-Irradiated Ginseng and Puffed Ginseng

Recently, two new ginseng processing methods have been developed. One method is microwave heating, which is a simple, efficient, and time-saving way to process ginsengs. Malonyl ginsenosides are much less stable than the corresponding neutral ginsenosides during microwave treatment [85]. When ginseng is subjected to microwave irradiation for 5 min, the ginsenosides Rh2 and Rg3 are produced through the degradation of Rc and Rd, respectively [86]. After microwave treatment, concentrations of ginsenosides Rg1, Re, Rb1, Rc, and Rb2, and Rd are reduced, whereas the concentrations of ginsenosides Rg3, Rk1, and Rg5 are increased. In addition, anticancer effects are enhanced by microwave processing; therefore, heat processing by microwave irradiation is considered a useful method to enhance the biological activities of ginseng by increasing the concentrations of minor ginsenosides [87]. The other novel method used to process ginseng is puffing. Puffed ginseng can be more easily dehydrated because of its more aerated and porous structure. Puffed ginseng produced by a rotary gun puffing machine contained greater crude saponin concentrations than non-puffed samples [88]. Due to the puffing pressure, minor ginsenosides (Rg3, F2, Rk1, and Rg5) are increased but the concentrations of major ginsenosides (Rb1, Rb2, Rc, Rd, Re, and Rg1) are reduced, which indicates puffing treatment significantly influences the ginsenoside composition [88,89].

#### 3.1.7. Transformation Pathways of Ginsenosides during Physical Processing

**Figure 3:**
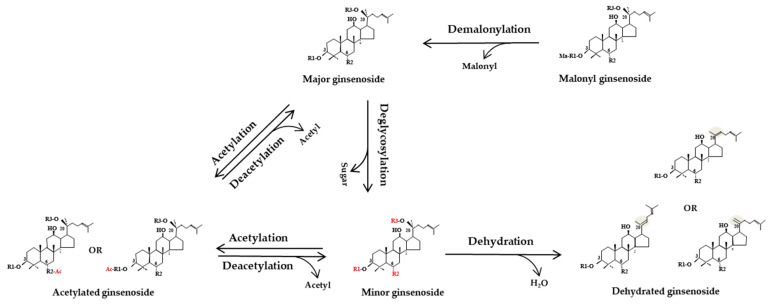
Ginsenoside transformation pathways during physical processing. Unstable malonyl ginsenosides may be demalonylated into corresponding ginsenosides; the sugar moiety attached to the C–3, C–6, or C–20 of major ginsenosides may be deglycosylated to produce minor ginsenosides; Dehydration may occur at C–20 and the resulting double bond is formed either between C–20 and C–21 or between C–20 and C–22, leading to positional and geometric isomerism.

The diverse ginsenoside profiles in physically processed ginseng products indicate that several transformation pathways are involved in altering the saponin composition (Figure 3). The pathways may include demalonylation of unstable malonyl-ginsenosides into the corresponding neutral ginsenosides during dehydrating, streaming, heating or other processing [14]. The sugar moiety attached to the C–3, C–6, or C–20 may also be deglycosylated to produce minor ginsenosides, such as Rb1, Rb2, Rc, Rd→Rg3, F2, Rh2; Re, Rf→Rg1, Rg2, F1, and Rh1 [62]. Dehydration may occur at C–20 and the resulting double bond is formed either between C–20 and C–21 or between C–20 and C–22, leading to positional and geometric isomerism: Rk1, Rg5 and Rz1 are dehydrated ginsenosides of Rg3 [90]; Rk2 and Rh3 are dehydrated ginsenosides of Rh2; Rh4 and Rk3 are dehydrated ginsenosides of Rh1; Rg6 and F4 are dehydrated from Rg2; Rs4 and Rs5 are dehydrated from Rs3 [28]; and Rg9 and Rg10 are dehydrated from [91]. Acetylation of several ginsenosides may generate acetylated ginsenosides, including the Rs4, Rs5, Rs6, and Rs7 acetylated ginsenosides of Rg5, Rk1, Rh4, and Rk3, respectively [81].

### 3.2. Ginsenoside Variation in Chemically Processed Ginseng 

To enhance the functionalities of ginseng, not only physical methods are used to process ginseng, but also chemical methods are applied, such as acid and alkaline hydrolysis.

#### 3.2.1. Ginsenoside Composition after Acid Hydrolysis

Recently, organic acids have been utilized to transform the major ginsenosides into minor ginsenosides and improve the biological activity of ginseng products. When white ginseng extract (WGE) was treated with 10% citric acid at 100 °C for 1 h, the concentrations of ginsenoside Rg3 were 10-fold greater than those of a non-treated sample, and the antitumor and antioxidant activities of the WGE increased [92]. Some studies also found that treatment of ginsenosides Rh1, Rh2, and Rg3 with 0.01% formic acid at 120 °C for 4 h resulted in the formation of ginsenosides Rk3 and Rh4, Rk1 and Rg5, and Rk2 and Rh3, respectively [93]. In addition, through acid hydrolysis, an addition reaction, and dehydration, formic acid promotes the transformation from ginsenosides Re, Rf, and Rg2 into several minor ginsenosides, i.e., F1, Rh1, Rf2, Rf3, Rg6, F4, and Rg9 [94,95]. Besides citric acid and formic acid, other acids, such as acetic acid, ascorbic acid, and lactic acid, can also be used in the chemical transformation of major ginsenosides into minor ginsenosides in ginseng samples [96,97].

#### 3.2.2. Ginsenoside Composition after Alkaline Hydrolysis

Alkaline hydrolysis is a method used to degrade ginsenosides under conditions of high temperature, high pressure, and high pH [98]. Two new dammarane-type triterpenes, namely ginsenoslaloside-I and 20(*S*)-ginsenoside-Rh1-60-acetate, together with twelve known compounds (including PPT, PPD, Rh16, Rh3, Rh1, F4, and Rk1) were isolated from the alkaline hydrolysate of total saponins after refluxing with 2 mol/L sodium hydroxide [99]. Total saponins in ginseng stems and leaves were hydrolyzed in a 2 mol/L sodium hydroxide aqueous solution suspended in a boiling water bath for 8 h, and a new compound, 20(*R*)-ginsenoside Rh19, was isolated and identified [100]. The alkaline cleavage products of ginsenoside Rb1 and Re were protopanaxadiol (PPD) and protopanaxatriol (PPT) after treatment with sodium methoxide at 85 °C for 8 h [101]. Moreover, ginsenoside Ro is mainly hydrolyzed at the C–28 ester bond after treatment at pH 13 and 60 °C, while zingibroside-R1 was the hydrolysate of ginsenoside Ro after alkali treatment [102].

### 3.3. Ginsenoside Variation in Biologically Processed Ginseng 

**Figure 4:**
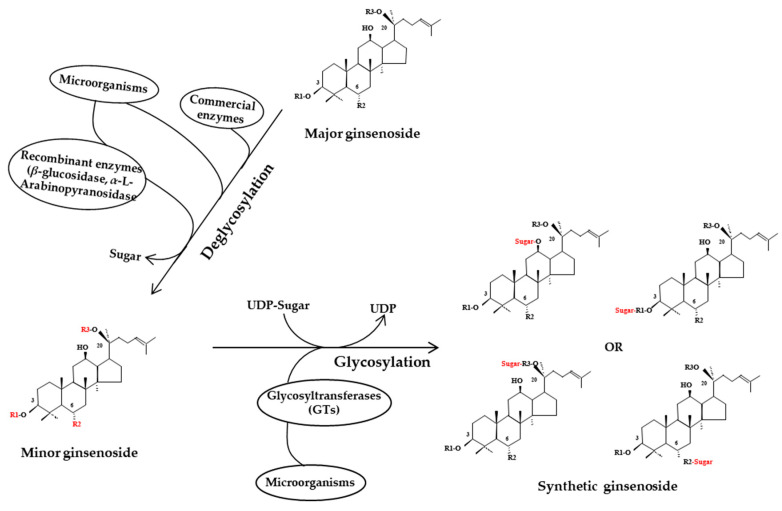
Ginsenoside transformation pathways during biological processing. The sugar moieties linked to C–3, C–6, or C–20 in the major ginsenosides are hydrolyzed by microorganisms, commercial enzymes, or recombinant enzymes; on the other hand, glycose molecules or activated sugars are conjugated to the aglycones of ginsenosides (glycosylation) by glycosyl hydrolase or glycosyltransferases, respectively. UDP, uridine diphosphate.

Biological processing methods are commonly used to process ginseng products given the strongly selective reactions, mild reaction conditions, and reduction in undesirable by-products, simple reprocessing, and other benefits. Fermentation using microorganisms or enzymes plays an important role in biological processing of ginseng. Fermented products have been found to produce more pharmacological effects than unfermented products, likely due to the effects of microbial transformation, enzyme conversion, and glycosyltransferase activity to change the ginsenoside composition (Figure 4). 

#### 3.3.1. Microbial Ginseng Fermentation

Fermentation of ginseng using microorganisms has been reported to improve the beneficial effects of ginseng because the microorganisms can convert glycosides to aglycones and/or produce their metabolites. One special ginsenoside, compound K (CK), is a bioactive ginsenoside that can be produced from ginsenoside Rb1and Rb2 by intestinal microflora [103]. Production of CK by physical or chemical methods has proven challenging. As a result, microorganisms have been widely used to increase the concentrations of compound K in ginseng products. When comparing fermented ginseng samples produced using various microorganisms, ginseng root fermented with *Lactobacillus brevis* (isolated from Kimchi) for five days produced the greatest concentrations of CK, and the resulting product may have potential as a functional food for treatment and prevention of various diseases [104]. After WG is fermented with *Bacillus* sp., the concentrations of Rd and Rg3 increase due to microbial transformation of Rb1 and Rc [105]. Fermentation of red ginseng with *Bifidobacterium* H–1 produces mainly CK, Rg3, and Rh2, and the product exhibits significantly better protection against ischemia-reperfusion brain injury than RG (which lacks Rh2 and CK). Kim et al. (2010) confirmed that red ginseng fermented with *L. plantarum* M1 is very useful for preparing minor ginsenoside metabolites (Rg3, CK, Rh1, Rh2) while being safe for foods [106,107]. Fermented black ginseng (FBG) is processed by repeated steaming and drying of fresh ginseng followed by fermentation with *Saccharomyces cerevisiae*, Fermentation of BG can produce more active ginsenosides and may have potential anti-wrinkle activity that could make it a desirable ingredient in cosmetics [108]; FBG may also protect cells against oxidative damage by scavenging ROS [109]. 

Microorganisms produce various enzymes, including β-glucosidase, β-glycosidase, β-galactosidase, and l-arabinofuranosidase, which may transform ginsenosides by cleaving the sugar moieties at C–3, C–6, and/or C–20. The transformation pathways for microbial fermentation [7,62,110] were as follows: Rb1→Rd→F2→CK; Rb1→Rd→Rg3→Rh2; Rb1, Rb2, Rc→Rd; Rc→Mc→CK; Rb2→compound O→compound Y→CK; Re→Rg2, Rh1; Re→Rg1→Rh1; and Rf→Rh1. 

#### 3.3.2. Enzymatically Fermented Ginseng

The enzymatic fermentation method produces ginseng products of greater purity with fewer by-products, and employs a short reaction cycle than the other methods for processing ginseng [111]. Different enzymes play a different role in hydrolyzing ginsenosides. Unlike microbial conversion methods, enzyme conversion methods can be performed at higher temperatures according to the enzyme characterization. Several commercial enzymes, such as Rapidase, Econase CE, Viscozyme, Ultraflo L, and Cytolase, have been used to ferment red ginseng extracts. The results indicate Rapidase and Cytolase can not only significantly increase the total amount of ginsenosides, but can also deglycosylate ginsenosides to Rg3 [112,113]. In addition, when five commercial enzymes were added to white ginseng extract for 60 h, the major ginsenosides were transformed into Rg3, F2, and CK. Cytolase PLC5 was selected as the most effective enzyme among enzymes tested because of the greater production of CK from WG extract [114]. In addition to the above enzymes, β-glucosidase, β-glycosidase, pectinase, cellulose, and naringinase have also been utilized for enzymatic conversion of ginseng. Pectinase, β-glucosidase, and β-glycosidase react with ginseng to produce CK [115], and cellulose has been used to generate Rg3 in WG extracts [116,117]. Moreover, cellulose mixed with naringinase increases conversion of Re and Rg1 into F1 [118]. 

Apart from commercial enzymes used for hydrolysis of ginsenosides, enzymes isolated from bacteria and fungi have also been recombined in host strains to enhance the enzyme activity. Several recombinant β-glucosidases show outstanding abilities for conversion of Rg3, Rh2, and CK by hydrolysis of the glucose at C–3 and C–20 [119,120,121]. Recombinant β-glucosidase (bgp 1) not only transforms Rb1 and Rd into Rg3, but also generates Rg2 and Rh1 from Re when reacted with ginseng leaf saponins [122]. Recombinant β-glucosidase isolated from *Aspergillus niger* is able to transform Rf into Rh1 [123]. In addition, dehydrogenases from *Cladosporium cladosporioide* and *L. brevis* was transferred CK and PPD to 3-oxo-CK and 3-oxo-PPD through ketonizations at C–3 position [124,125]. Moreover, recombinant α-l-arabinopyranosidase from *Blastococcus saxobsidens* mediates enzymatic conversion of Rb2, compound O, and compound Y into Rd, F2, and CK, respectively [126]. In the future, additional ginseng products produced by enzymatic processing could be developed based on the specific activities of particular recombinant enzymes. 

In addition to enzymatic deglycosylation by hydrolyzing the sugar moieties linked to C–3, C–6, or C–20, glycosylation catalyzed by glycosyltransferases (GTs) also plays a key biological role as the final step in ginseng saponin synthesis. The GT enzymes transfer glycosyl residues from activated sugars to the aglycones of ginsenosides, thus regulating the properties of ginsenosides, such as bioactivity, solubility and stability [127]. A novel α-glycosylated ginsenoside F1 (G1–F1) was generated from transglycosylation reactions of dextrin and ginsenosiede F1 by a cyclodextrin glucanotransferase [128]. Two novel ginsenosides, glucosyl ginsenoside Rh2 and diglucosyl ginsenoside Rh2, have been produced using recombinant GTs from *Lactobacillus rhamnosus* and show greater anticancer activity than Rh2 [129]. Synthetic ginsenoside Rd12 was synthesized using UDP-glycosyltransferase from *Bacillus subtilis* 168 [130]. Moreover, mass production of the rare ginsenoside Ia from F1 using recombinant UDP-glycosyltransferase isolated from *B. subtillis* has been reported and ginsenoside Ia demonstrates superior melanogenesis inhibitory ability in B16BL6 cells compared to F1 [131]. The structures of the ginsenoside presented in this review are listed in Table 2.

## 4. Conclusions

Ginseng is a traditional medicinal herb and is consumed worldwide due to its broad pharmacological activity. Ginsenosides are the main active compounds in ginseng and have unique biological activities and medicinal values. There are various processed ginseng products (such as fresh ginseng, white ginseng, boiled ginseng, steamed ginseng, acid-processed ginseng, and fermented ginseng). The variation in the ginsenoside compositions of ginseng products may be a function of the processing method and may underlie the differing pharmacological properties of ginseng products. The diversity of the main ginsenosides in different ginseng species may also reflect the environments and regions where ginseng plants are grown. In particular, PPT- and PPD-type ginsenosides are abundant in *Panax ginseng*, *P. quinquefolius,* and *P. notoginseng*; and *P. japonicasi,* while *P. vietnamensis* contains OT-type ginsenosides as the main saponins. In contrast, OA-type saponins are rich in other species of ginseng (*P. zingiberensi, P. stipuleanatus, P. bipinnatifidus*, and *P. sokpayensis*). In addition, chemical reactions during the physical and chemical processing of ginseng, such as demalonylation, deglycosylation, acetylation, hydrolysis, addition reactions, and dehydration, may result in the generation of more biologically active saponins compared to fresh ginseng. Moreover, several minor and unnatural ginsenosides are formed during biological processing of ginseng by enzyme/microbial biotransformation or glycosylation catalyzed by various glycosyltransferases. Processed ginseng products may contain a diverse array of main ginsenosides and biological activities; therefore, future studies addressing the use of a combination of several processing methods to increase the concentrations of minor ginsenosides may enhance the pharmaceutical value of the resulting ginseng products.

## Figures and Tables

**Table 1:** Ginsenosides types in various *Panax* species.

Species	Common Name	Geographical Distribution	Main Saponin Types	Distinctiveness	Ref.
*P. ginseng*	Korean ginseng	Asian countries	PPT and PPD	G–Rf (PPT)and G–Rs1 (PPD)	[24,25,26,28,29,30]
*P. quinquefolius*	American ginseng	America	PPT and PPD	P–F11 (OT)	[26,29,31]
*P. notoginseng*	Chinese (Sanchi) ginseng	China	PPT and PPD	Noto–R1 (PPT)	[26,29]
*P. japonicas*	Japanese ginsengor Ye–sanchi	China and Japan	OT	Yesanchinosides (OT)	[32,33,34,35]
*P. vietnamensis*	Vietnam ginseng	Vietnam	PPT, PPD, and OT	Majon–R2 (OT)	[36,37,38]
*P. zingiberensis*	Ginger ginseng or Myanmar ginseng	China	OA and PPT	-	[39,40,41,42]
*P. stipuleanatus*	Pingpien ginseng	China and Vietnam	OA	-	[43,44,45]
*P. bipinnatifidus*	Feather-leaf bamboo ginseng	China, Eastern Himalayas, and Nepal	OA	-	[46,47,48]
*P. sokpayensis*	-	India	PPT and PPD	-	[47]
*P. omeiensis*	Omei ginseng	China, Eastern Himalayas, and Nepal	-	-	[11,48]
*P. pseudoginseng*	Himalayan ginseng	China, Eastern Himalayas, and Nepal	PPT, PPD, and OA		[11,48,49,50,51]
*P. assamicus*	-	India and West Bengal	-	-	[52,53,54]
*P. shangianus*	-	China	-	-	[11,54]
*P. sinensis*	-	China	-	-	[11,55,56]
*P. trifolius*	Dwarf ginseng	Ohio andPennsylvania	PPT, PPD, and OA	-	[48,57,58]
*P. variabilis*	-	China and India	-	-	[11,54]
*P. wangianus*	Narrow-leaved pseudoginseng	China and India	-	-	[48,59,60,61]

Notes: PPT, protopanaxatriol; PPD, protopanaxadiol; OT, ocotillol; OA, oleanolic acid; G, ginsenoside; P, pseudoginsenoside; Noto, notoginsenoside; Majon, majonoside.

**Table 2:** Structures of ginsenosides present in this review.

Types	Name	R1	R2	R3	Remark
PPD 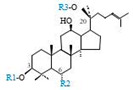	G–Rb1	glc(2–1)glc	H	glc(6–1)glc	Major ginsenoside
	G–Rb2	glc(2–1)glc	H	glc(6–1)ara(p)	Major ginsenoside
	G–Rc	glc(2–1)glc	H	glc(6–1)ara(f)	Major ginsenoside
	G–Rd	glc(2–1)glc	H	glc(6–1)	Major ginsenoside
	Ma–Rb1	glc(2–1)glc(6)Ma	H	glc(6–1)glc	Malonyl ginsenoside
	Ma–Rb2	glc(2–1)glc(6)Ma	H	glc(6–1)ara(p)	Malonyl ginsenoside
	Ma–Rc	glc(2–1)glc(6)Ma	H	glc(6–1)ara(f)	Malonyl ginsenoside
	Ma–Rd	glc(2–1)glc(6)Ma	H	glc(6–1)	Malonyl ginsenoside
	G–Rb3	glc(2–1)glc	H	glc(6–1)xyl	Ginsenoside
	G–Ra3	glc(2–1)glc	H	glc(6–1)glc(3–1)xyl	Ginsenoside
	Noto–R4	glc	H	glc(6–1)glc(6–1)xyl	Notoginsenoside
	G–Rs1	glc(2–1)glc(6)Ac	H	glc(6–1)ara(p)	Acetylated ginsenoside
	G–Rs3	glc(2–1)glc(6)Ac	H	H	Acetylated ginsenoside
	G–F2	glc	H	glc	Minor ginsenoside
	G–Rg3	glc(2–1)glc	H	H	Minor ginsenoside
	G–Rh2	glc	H	H	Minor ginsenoside
	G–Mc	H	H	glc(6–1)ara(f)	Minor ginsenoside
	G–Compound O	glc	H	glc(6–1)ara(p)	Minor ginsenoside
	G–Compound Y	H	H	glc(6–1)ara(p)	Minor ginsenoside
	G–Compound K	H	H	glc	Minor ginsenoside
PPT 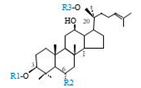	G–Re	H	Oglc(2–1)rha	glc	Major Ginsenoside
	G–Rg1	H	Oglc	glc	Major Ginsenoside
	G–Rf	H	Oglc(2–1)glc	H	Major Ginsenoside
	G–Rg2	H	Oglc(2–1)rha	H	Minor Ginsenoside
	G–Rh1	H	Oglc	H	Minor Ginsenoside
	G–F1	H	OH	glc	Minor Ginsenoside
	G–F3	H	OH	glc(6–1)ara(p)	Minor Ginsenoside
	Noto–R1	H	Oglc(2–1)xyl	glc	Notoginsenoside
C17SCV–1 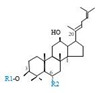	G–Rg5	glc(2–1)glc	H		Dehydrated ginsenoside
	G–F4	H	Oglc(2–1)rha		Dehydrated ginsenoside
	G–Rh4	H	Oglc		Dehydrated ginsenoside
	G–Rh3	glc	H		Dehydrated ginsenoside
	(20E)–G–Rg9	H	Oglc(2–1)glc		Dehydrated ginsenoside
	G–Rs4	glc(2–1)glc(6)Ac	H		Acetylated ginsenoside
C17SCV–2 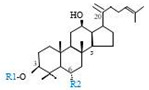	G–Rs6	H	Oglc(6)Ac		Acetylated ginsenoside
	G–Rk1	glc(2–1)glc	H		Dehydrated ginsenoside
	G–Rk3	H	Oglc		Dehydrated ginsenoside
	G–Rg6	H	Oglc(2–1)rha		Dehydrated ginsenoside
	G–Rk2	glc	H		Dehydrated ginsenoside
	G–Rg10	H	Oglc(2–1)glc		Dehydrated ginsenoside
	G–Rs5	glc(2–1)glc(6)Ac	H		Acetylated ginsenoside
	G–Rs7	H	Oglc(6)Ac		Acetylated ginsenoside
C17SCV–3 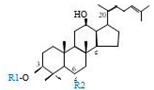	G–Rz1	glc(2–1)glc	H		Dehydrated ginsenoside
	(20Z) –G–Rg9	H	Oglc(2–1)glc		Dehydrated ginsenoside
	G–Rh16	glc	H		Dehydrated ginsenoside
C17SCV–4 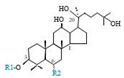	G–Rf2	H	Oglc(2–1)rha		Hydration addition reaction
	G–Rf3	H	Oglc(2–1)glc		Hydration addition reaction
OT 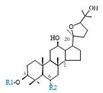	P–F11	H	Oglc(2–1)rha		Pseudoginsenoside
	Majon–R1	H	Oglc(2–1)glc		Majonoside
	Majon–R2	H	Oglc(2–1)xyl		Majonoside
	Vina–R1	H	O6–Ac–glc(2–1)rha		Vinaginsenoside
	Vina–R2	H	O6–Ac–glc(2–1)xyl		Vinaginsenoside
OA 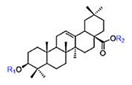	G–Ro	glcUA(2-1)glc	-glc		Ginsenoside
	CS–IV	glcUA(4-1)ara	-glc		Chikusetsu saponin
	CS–IVa	glcUA	-glc		Chikusetsu saponin
	Zing–R1	glc(2-1)glc	H		Zingibroside
	Spina-A methyl ester	6-CH3-glcA(3-1)glc	H		Spinasaponin
	Stipul–R1	[3-O-glc]-glcA(4-1)ara(f)	H		Stipuleanoside
	Stipul–R2	[3-O-glc]-glcA(4-1)ara(f)	-glc		Stipuleanoside
	Pseudo–RP1	glcA(2-1)xyl	H		Pesudoginsenoside
	Pseudo–RT1	glcA(2-1)xyl	-glc		Pesudoginsenoside
	Bifi-A	-6-CH3-glcA(2-1)ara(p)	H		Bifinoside
	Bifi-B	-6-CH3-glcA(2-1)glc(6-1)xyl	H		Bifinoside
	Bifi-C	-6-CH3-glcA [3-ara(p)]glc(2-1)xyl	-glc		Bifinoside
Dammarane 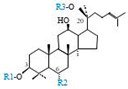	Glucosyl-G- Rh2	-glc(6-1)glc	H	H	Synthetic ginsenoside
	Diglucosyl-G-Rh2	-glc(6-1)glc(6-1)glc	H	H	Synthetic ginsenoside
	G-Ia	-glc	-OH	glc	Synthetic ginsenoside
	α-Glycosylated-G F1	H	H	-glc(1-2)α-D-glucopyranoside	Synthetic ginsenoside
Miscellaneous-1 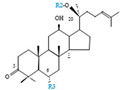	3-oxo-CK	-	H	-glc	Ketonization ginsenoside
	3-oxo-PPD	-	H	H	Ketonization ginsenoside
Miscellaneous-2 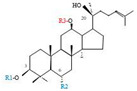	G-Rd12	-glc(2-1)glc	H	-glc	Synthetic ginsenoside

Note: PPT, protopanaxatriol; PPD, protopanaxadiol; C17SCV, C17 side-chain varied; OT, ocotillol; OA, oleanolic acid; SG, synthetic ginsenoside; G, ginsenoside; Noto, notoginsenoside; glc, glucose; ara, arabinose; p, pyran; f, furan; rha, rhamnose; xyl: xylose; Ma, malonyl; Ac, acetyl; Majon, majonoside; Vina, vinaginsenoside; CS, chikusetsu saponin; Zing, zingibroside; Spina, spinasaponin; Stipil, stipuleanoside; Pesudo, pesudoginsenoside; Bifi, bifinoside, 3-oxo, ketonizing the hydroxyl group at C-3.
